# No evidence for direct thermal carryover effects on starvation tolerance in the obligate blood‐feeder, *Glossina morsitans morsitans*

**DOI:** 10.1002/ece3.10652

**Published:** 2023-10-18

**Authors:** Hester Weaving, Jennifer S. Lord, Lee Haines, Sinead English

**Affiliations:** ^1^ School of Biological Sciences University of Bristol Bristol UK; ^2^ Department of Vector Biology Liverpool School of Tropical Medicine Liverpool UK; ^3^ Department of Biological Sciences University of Notre Dame Notre Dame Indiana USA

**Keywords:** carryover effect, climate change, developmental plasticity, heat stress, starvation tolerance, tsetse

## Abstract

Thermal stress during development can prime animals to cope better with similar conditions in later life. Alternatively, negative effects of thermal stress can persist across life stages and result in poorer quality adults (negative carryover effects). As mean temperatures increase due to climate change, evidence for such effects across diverse taxa is required. Using *Glossina morsitans morsitans*, a species of tsetse fly and vector of trypanosomiasis, we asked whether (i) adaptive developmental plasticity allows flies to survive for longer under food deprivation when pupal and adult temperatures are matched; or (ii) temperature stress during development persists into adulthood, resulting in a greater risk of death. We did not find any advantage of matched pupal and adult temperature in terms of improved starvation tolerance, and no direct negative carryover effects were observed. There was some evidence for indirect carryover effects—high pupal temperature produced flies of lower body mass, which, in turn, resulted in greater starvation risk. However, adult temperature had the largest impact on starvation tolerance by far: flies died 60% faster at 31°C than those experiencing 25°C, consequently reducing survival time from a median of 8 (interquartile range (IQR) 7–9) to 5 (IQR 5–5.25) days. This highlights differences in temperature sensitivity between life stages, as there was no direct effect of pupal temperature on starvation tolerance. Therefore, for some regions of sub‐Saharan Africa, climate change may result in a higher mortality rate in emerging tsetse while they search for their first blood meal. This study reinforces existing evidence that responses to temperature are life stage specific and that plasticity may have limited capacity to buffer the effects of climate change.

## Graphical abstract

We find that adult temperature had the most pronounced effect on starvation tolerance in tsetse. There were no direct carryover effects of pupal temperature, showing that starvation responses to temperature are life‐stage specific. Therefore, rising temperatures due to climate change are likely to exert the greatest effect on emerging adult tsetse, forcing flies to locate their first blood meal more rapidly. Photo credit: Lee Haines.

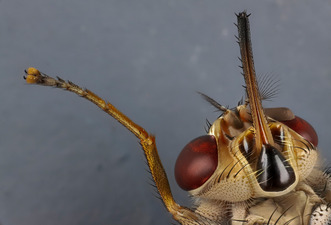

## 1 INTRODUCTION

Many animals can modify their phenotypes in response to the environment experienced during development, often in a way that provides a fitness benefit in adulthood (Fawcett & Frankenhuis, 2015; West‐Eberhard, 2002). This adaptive developmental plasticity may be an important response to heat stress given rising mean temperatures due to climate change, especially as research indicates that insects tend to be most plastic during developmental stages, rather than adulthood (Pottier et al., 2022; Weaving et al., 2022). Phenotypic changes can be adaptive when offspring and adult thermal environments are matched, or when variability is predictable, such as seasonal variation (Monaghan, 2008). However, these anticipatory phenotypic changes can have a deleterious effect if the environment becomes mismatched, for example, as seasons advance due to climate change, or as conditions become more unpredictable and extreme (Parmesan, 2006). Thermal stress during development can also be harmful, where deleterious effects extend from juvenile stages into adulthood, which can result in the emergence of poor‐quality adults (Kingsolver & Huey, 2008).

Insects are a good model for the study of developmental plasticity due to their life histories with multiple distinct stages, and trackable, short life spans (English & Barreaux, 2020). Adaptive developmental plasticity, where matching juvenile and adult temperatures provides a fitness benefit, has been previously observed in insects. For example, high developmental temperature leads to lighter adult pigmentation in the harlequin bug (*Murgantia histrionica*), and fewer dark spots in ladybirds (*Harmonia axyridis*), which subsequently reduces the risk of overheating (Michie et al., 2011; Sibilia et al., 2018). Similarly, heat stress during development in red flour beetles, *Tribolium castaneum*, improves heat shock tolerance in adults (Scharf et al., 2014). In contrast, if juvenile and adult environments are mismatched, plasticity can become deleterious. For example, the tropical butterfly, *Bicyclus anynana*, is seasonally polymorphic with camouflage suited to either the warm, wet season or the dry, cool season. When the conspicuous, wet season form is released in the dry season, these butterflies have lower rates of survival compared to the resident form (Brakefield & French, 1999; Brakefield & Reitsma, 1991). Unseasonable temperatures caused by climate change could therefore trigger a mismatch of wing pattern to season, thus resulting in greater predation (De Jong et al., 2010).

Deleterious plasticity can also occur when unfavourable conditions during development result in reduced fitness in the adult stage, which we define here as negative carryover effects (although note that carryover effects can be defined as effects which increase fitness too, see O'connor et al., 2014). Warmer developmental temperatures often result in a smaller adult body size, known as the temperature‐size rule, which tends to be related to lower fecundity, mating success and survival (Kingsolver & Huey, 2008). Heat stress during development can additionally cause direct damage to reproduction, as demonstrated in several *Drosophila* species. When larvae develop at their upper thermal limit, emerging adult males have reduced sperm mobility and smaller testis length, and females have fewer ovarioles and reduced fertility (Porcelli et al., 2017; Sisodia & Singh, 2009).

Studies have typically focused on how heat stress during development affects later adult thermal tolerance traits, but some have considered other fitness‐related traits such as starvation tolerance. Starvation tolerance is a useful fitness proxy because many organisms face periods without food, particularly those where prey are temporally or spatially unpredictable. As metabolic rate increases with temperature, climate change puts ectothermic animals at risk of starvation if they cannot increase feeding rate to meet metabolic demands. Starvation tolerance has a plastic component, related to the ability of an organism to reduce its metabolic rate, the quantity of energetic reserves held, and the minimum amount of these reserves required for survival (Rion & Kawecki, 2007). Ectotherms that have greater plasticity of starvation tolerance are likely to be more resilient to climate change (Norin & Metcalfe, 2019).

Cross‐tolerance, where exposure to one environmental stressor results in increased resistance to another, has been demonstrated for starvation tolerance in several insect species for environmental variables such as heat, cold and humidity (Bauerfeind et al., 2014; Bubliy et al., 2012; Gotcha et al., 2018; Kalra et al., 2017). However, cross‐tolerance is not universal to all combinations of stressors as some studies find no association or a negative association. Studies that investigate cross‐tolerance of developmental plasticity in starvation tolerance also find mixed results. In the red flour beetle, *T. castaneum*, adults raised at high developmental temperature had better tolerance to starvation as adults, as well as improved heat and cold tolerance (Scharf et al., 2014). However, the difference in starvation tolerance was greater when adults were maintained at a benign temperature, rather than when development and adult temperatures were matched. Similarly, in the butterfly *B. anynana*, a high developmental temperature increased starvation tolerance in adults, but this result was dependent on polyphenic morph and sex (Pijpe et al., 2007). Clearly, thermal developmental plasticity of starvation tolerance is complex and further studies are needed, particularly using a greater diversity of species with varying life histories.

Starvation tolerance is relevant to blood‐feeding vectors as vertebrate blood is a temporally and spatially unpredictable food source. This is particularly true for obligate blood feeders, where the time between blood meals is critical to survival as they cannot rely on other sources of sustenance. As temperatures increase due to climate change, host availability may shift (Dawe & Boutin, 2016; Simon et al., 2014) and higher metabolic demands of ectothermic blood feeders may lead to behavioural changes, such as increased bite rate (Rogers & Packer, 1993). Studies have also shown that a period of starvation can increase competency to infection in vectors such as mosquitoes and tsetse flies (Gingrich et al., 1982; Gooding, 1988; Herd et al., 2021). Therefore, research into how starvation tolerance is affected by temperature can have important epidemiological implications.

Tsetse (*Glossina* spp.) are insect vectors of human and animal African trypanosomiasis, which are prevalent diseases throughout sub‐Saharan Africa (Buxton, 1955). Trypanosomiases are responsible for over 200,000 disability‐adjusted human life years and losses of approximately 1 million cattle per year (Cecchi & Mattioli, 2009; Shaw, 2004). Both male and female flies feed exclusively on vertebrate blood so may undergo extended periods without food due to the spatial and temporal unpredictability of vertebrate animals (Lehane, 2005; Lord et al., 2017). Newly emerged unfed flies, hereafter teneral flies, are at particular risk of starvation as they rely solely on energy reserves provided by the mother until they locate their first blood meal. This is due to adenotrophic viviparity, an unusual method of reproduction for insects, where mothers nourish one offspring at a time in utero with a milk‐like secretion (Benoit et al., 2015; Haines et al., 2020). The offspring is deposited as a late‐stage larva that rapidly burrows into the ground to pupate, and remains there until emergence around a month later (Buxton, 1955). Teneral flies must then rapidly locate their first blood meal or risk starvation and dehydration (Hargrove, 2004; Lord et al., 2017; Phelps & Clarke, 1974).

In the laboratory, relationships between temperature and development rates, body size, lipid consumption and pupal mortality have been well documented for *Glossina morsitans morsitans* Westwood (Bursell, 1960; Hargrove & Vale, 2020; Phelps, 1973; Phelps & Burrows, 1969). As pupal temperature increases, development times become shorter and resulting adults are smaller (Bursell, 1960). The total lipid consumed during pupation is lowest at 25°C, and thus the reserves available to a teneral fly decrease above this temperature. At temperatures above 32°C, increased pupal mortality rates result from direct effects of temperature, rather than from lipid consumption alone (Hargrove & Vale, 2020). Developmental plasticity has also been investigated in tsetse, where changes to the temperature during development results in plasticity of lower thermal limits and water loss rate in adults, but no effect is seen on upper thermal limits (Terblanche & Chown, 2006). Despite the importance of temperature on the quality of emerging teneral flies, the effect of developmental temperature on adult starvation tolerance remains unknown in tsetse.

Here, we investigate whether pupal or adult temperature stress influences starvation tolerance in tsetse (*G. m. morsitans*). The aim of the study was to test whether adaptive developmental plasticity allows flies to survive for longer under food deprivation when pupal and adult temperatures are matched, or if negative carryover effects from a high temperature during development reduces starvation tolerance in emerging adults. We also measured the effects of developmental temperature on adult size, and the relationship between size and starvation tolerance, as a potential mechanism linking pupal temperature and adult survival.

## 2 METHODS

### 2.1 Colony maintenance

*Glossina morsitans morsitans* pupae were collected from an established colony at the Liverpool School of Tropical Medicine (LSTM) on 14 and 15 November 2019. The colony is maintained at 25 ± 2°C, 68%–79% relative humidity (RH) and a 12:12 h light: dark cycle, and fed on defibrinated horse blood (TCS Biosciences) three times a week. Pupae were held 5–48 h after being deposited under the above conditions until transport to the University of Bristol. For each pupa, maternal age was known ±1 week, ranging from 3 to 9 weeks old. Misshapen and undersized pupae were not included in the study.

### 2.2 Experimental design

**FIGURE 1:**
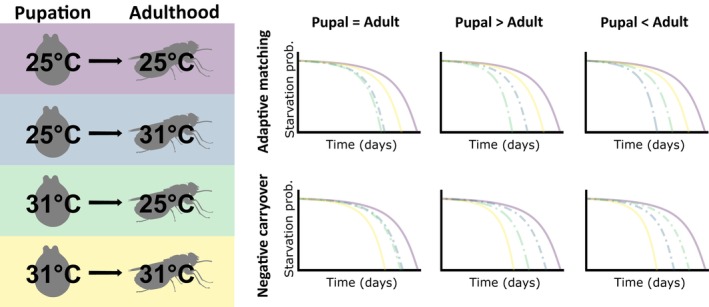
Overview of the experimental design and predicted outcomes. Pupae were kept at either constant 25°C or 31°C for development and transferred to either a matched (solid) or unmatched (dot‐dashed) temperature upon emergence for adulthood. The time until death was recorded in the absence of blood meals. Predictions are given for the relationship between the probability of starvation and time (days) under the hypotheses of either adaptive matching or negative carryover effects. Predictions are given for: an equal effect of pupal and adult temperature on starvation tolerance, or an unequal effect, that is, Pupal > Adult or Adult < Pupal.

Pupae (*N* = 440) were divided between four treatment groups (Figure 1). Pupae from each collection date and maternal age group were divided equally between the groups as there is a relationship between pupal mass and maternal age (Lord et al., 2021; Table S1). Each pupa was transferred into a 50 mL falcon tube with a conical base (Fisher Scientific, Loughborough) drilled with 2 mm holes in the centre of each lid to allow for gas exchange. Treatment groups followed a full factorial design. Half of the pupae were maintained at 25°C and half at 31°C. Upon adult emergence, half of the flies were moved to the alternate treatment, and the other half remained at the same temperature. This gave four treatments in total: two where the pupal and adult stages were matched (25°C/25°C or 31°C/31°C) and two where the pupal and adult stages were mismatched (25°C/31°C or 31°C/25°C). The control treatment of 25°C was chosen as a baseline as the minimum quantity of lipid is used at this temperature during the pupal stage, and as this is the temperature at which the tsetse colony at Liverpool School of Tropical Medicine is maintained. A temperature of 31°C was used as a heat treatment as it is near to the upper temperature at which pupation will be completed (32°C), but few deaths occur due to the direct effects of heat stress (Hargrove & Vale, 2020). Tsetse regularly experiences these temperatures in the field. For example, see daily temperature data collected at Rekomitjie Research Station in Mana Pools National Park in Supplementary data for Lord et al. (2018).

The 25°C treatment was maintained in a Reftech room (Reftech, Sassenheim, The Netherlands), and the 31°C treatment in an incubator (Snijders Labs, Tilburg, The Netherlands). Flies were maintained at 60% RH, in a 8:16 h light: dark cycle. Temperature and humidity were recorded using iButton data loggers (Mouser Electronics, High Wycombe) placed inside an empty falcon tube. iButtons recorded actual mean temperatures (±SD) of 24.9 ± 0.8°C, and 31.3 ± 0.4°C. The 25°C and 31°C treatments were 56.1 RH ± 1.4% and 55.9 RH ± 0.9%, respectively. Relative humidity differed by 8%–19% from the LSTM colony conditions due to other experiments running in the communal Reftec room concurrently. Approximately 10% reduction in emergence has been found between 80% RH and 60% RH (Bursell, 1958). Light: dark cycle was altered from colony conditions from 9 AM–9 PM to 9 AM–5 PM. Altering dusk does not affect flight patterns in *G. m. morsitans* so this alteration should not affect tsetse activity (Brady & Crump, 1978). Humidity and light: dark cycle were matched between treatments to eliminate potential confounding variables in each group.

### 2.3 Data collection

#### 2.3.1 Development time

Pupae were checked daily between 09:00 and 10:00 for adult emergence. Pupal development time was recorded in days, and emerging flies were either returned to the original temperature treatment or moved to the contrasting treatment. Flies were sexed upon emergence. Pupae were assumed inviable if they had not emerged by the end of the 47 day experiment as development takes approximately 30 days at 25°C degrees (Phelps & Burrows, 1969).

#### 2.3.2 Pupal and adult size and mass

We measured both mass and size to account for the direct effects of temperature on body mass or size, and to ascertain whether they affect starvation tolerance. Both mass and size measurements were taken as mass indicates resource use throughout the experiment, but adult size is influenced by developmental temperature, becoming fixed at emergence. Wet mass measurements were taken at three stages: pupal mass (approx. 60 h after larviposition), adult mass at emergence (within 24 h), and mass at death (within 24 h). Pupal mass indicates the reserves provided maternally at the start of the experiment, adult mass is indicative of the effect of pupal temperature on reserves and mass at death indicates the effect of pupal and adult temperature on reserves. Mass loss was calculated for the pupal and adult stages (by subtraction of the start and end mass) to give a rough estimate of energy reserve use and water loss (Brady, 1975). A Sartorius CPA26P balance (Göttingen, Germany), accurate to 2/10,000 mg, was used to take the mass measurements. Adult flies were kept at room temperature (~20°C) when weighed, and the process lasted no longer than 25 min.

Wing length – a proxy for adult size – was measured by removal of the left wing after a fly had died. The hatchet cell section of the fourth longitudinal vein was selected for measurement (Figure S1; Jackson, 1946). Images were taken with a Leica MZ21 camera microscope (Wetzlar, Germany) and LAS V3.8. The image (calibrated using a 1 mm graticule) was later analysed using ImageJ (Version 1.52a) to measure the wing vein in mm. Wing measurements could not be obtained for 43/428 adults because these emerged adult flies were unable to unfurl their wings (see Results section for details on how these were distributed across the temperature treatments).

#### 2.3.3 Starvation tolerance

Teneral adult fly mortality was recorded daily between 09:00 and 10:00 h by flicking the tube in which the fly was held to test for movement. If no movement was observed, the fly was considered dead and the number of days since emergence was recorded as the time until death. Here, we consider the number of days between adult emergence until death as an indicator of starvation tolerance, but we note that, since tsetse consume only blood, death may have been due to both dehydration and starvation. Additionally, we note that there is growing awareness and evidence that invertebrates experience pain and suffering. Therefore, we are reviewing our protocols, such as this starvation assay, to limit suffering and encourage other researchers to do so.

### 2.4 Data analysis

#### 2.4.1 Development time

All data analysis was completed in R Studio (Version 3.5.1; R Core Team, 2023). The effect of pupal temperature, sex and mass (and the two‐way interactions between these variables) on development time was determined by Generalised Least Squares due to unequal variances across treatment groups. We used the nlme package (Version 3.1‐162) and fit the model by the residual maximum likelihood method. Development time was log‐transformed so that the model residuals were normally distributed. The final model was determined by comparing AIC and deviance between models, as well as examining residuals for normality. The ‘drop one’ method was used whereby, starting from a maximal model, each term is excluded and removed if it does not significantly improve model fit (Bradburn et al., 2003). Post hoc analyses were completed by Tukey Honest Significant Differences using the emmeans package (Version 1.8.5). Adults that did not emerge from the puparium were not included in the models (*N* = 12; see Results for a breakdown depending on temperature treatment).

#### 2.4.2 Adult size and lifetime mass loss dynamics

The effect of pupal temperature and sex on adult size (either using wet mass or wing vein length) was determined by a linear model, controlling for maternal age as a covariate (as it has been shown to affect offspring size [Lord et al., 2021]). Variables with no significant effect were removed using the ‘drop one’ method. Post hoc analysis was completed as above. Mass loss through pupation and adulthood (until death) was also investigated by two generalised linear modes. Pupal mass loss data were not normally distributed, so the log link function was used. The effects of temperature, sex and their interaction, on mass loss were tested using the ‘drop one’ method. All model residuals were normally distributed.

#### 2.4.3 Starvation tolerance and risk

A Cox proportional hazards model was used to determine how starvation tolerance differed between the four temperature treatments, and whether pupal mass or sex had an effect. We used packages ‘survivalAnalysis’ (Version 0.3.0), ‘survival’ (Version 3.2.13) and ‘survminer’ (Version 0.4.9; Therneau & Grambsch, 2000). Temperature, sex and pupal mass and interactions between these variables were investigated. Variables with no significant effect were removed using the ‘drop one’ method. Hazard ratios refer to the risk of starvation where ratios of greater than 1 suggest a higher risk of death, and below 1, a lower risk of death compared to the reference group. We use pupal mass in these models as it is independent of the temperature treatments so represents differences in size before the experiment began. Three individuals were not included in the analysis as they died within 24 h of emergence, that is, before transfer into the adult temperature (due to failure to leave the pupal case). Post hoc analyses were completed by Tukey Honest Significant Differences using the emmeans package.

Based on reviewer comments, we also ran a path analysis in order to ascertain whether pupal temperature indirectly affected starvation tolerance through its effect on adult mass, in addition to the direct effects of pupal and adult temperature. We modelled the effect of pupal temperature and pupal mass on adult mass and the effect of pupal temperature, adult temperature, pupal mass and adult mass on starvation tolerance. We ran the model using packages lavaan (Version 0.6‐15) and semplot (Version 1.1.6) for data visualisation.

## 3 RESULTS

### 3.1 Development time is shorter at high temperatures and in females

**FIGURE 2:**
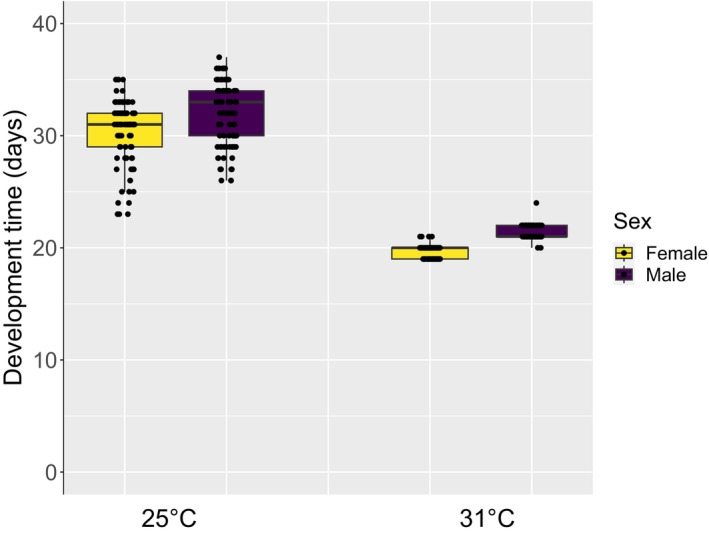
Pupal development time (days) for tsetse (*G. m. morsitans*) maintained at a constant temperatures of 25°C or 31°C. Median values and interquartile ranges are displayed with data split into females and males.

Pupal development time was 10.8 days shorter when pupae were kept at 31°C compared to 25°C (gls: *F* = 4160, df = 1, *p* < .001), and females developed 1.6 days more quickly than males, as expected based on previous studies (gls: *F* = 515, df = 1, *p* < .001, Tables S2, S3; Figure 2). An interaction between sex and temperature showed that the difference in development time between males and females was slightly greater at higher temperatures (gls: *F* = 7.7, df = 1, *p* < .001; Tables S3, S4). Pupal mass had no effect on development time (gls: *F* = 3.3, df = 1, *p* = .07; Table 2; Tables S2, S3). Only two individuals failed to emerge in the 25°C treatment, whereas there were 10 inviable pupae in the 31°C treatment. Of the 10% (*N* = 43/428) of individuals that emerged with deformed wings, almost all (*N* = 42/43) belonged to the high‐temperature treatment, and most were male (female:male, 15:28). All individuals (*N* = 3/428) that emerged but failed to leave the puparium were from the 31°C treatment.

### 3.2 High pupal temperature produces smaller adults

Initial pupal mass before temperature treatment did not differ between treatments or sexes. Controlling for maternal age and sex, emerging adults were smaller when pupae developed at 31°C as evidenced by adult mass (lm: SS = 150, df = 1, *p* < .001) and wing vein length (lm: SS = 0.004, df = 1, *p* < .001; Table S5). Individuals maintained at 31°C during pupation weighed on average 1.1 mg less (5%) at emergence, and their wing veins were 0.006 mm shorter (0.4%) than those maintained at 25°C. Across both temperature treatments, adult males weighed 0.80 mg less than females (3%) and had 0.16 mm shorter wing veins (10%).

### 3.3 Mass loss is greater at high temperature and during adulthood

**FIGURE 3:**
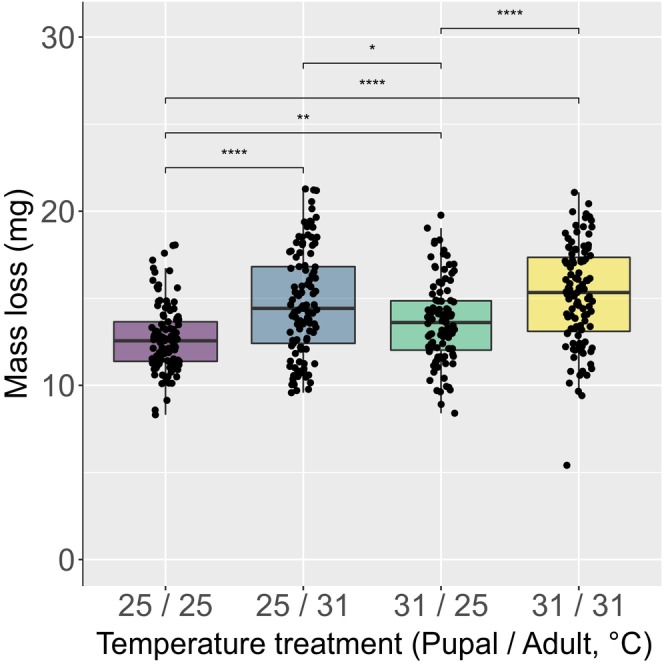
Total mass loss (mg) of tsetse (*G. m. morsitans*) under four temperature regimes (Pupal/Adult) of constant 25°C or 31°C. Shown are the median, interquartile range and confidence interval (boxes and lines) as well as the individual data (black points). *p < .05; **p < .01; ***p < .001; ****p < .0001.

Mass loss in pupae (glm: χ^2^ = 65.5, df = 1, *p* < .0001) and adults (glm: χ^2^ = 56.7, df = 3, *p* < .001) exposed to 31°C was greater than those kept at 25°C (Tables S6, S7; Figure 3). Rate of adult mass loss was approximately 10‐fold greater than the rate of pupal mass loss (Table S6). Across both temperature treatments, males lost more mass than females during pupal development (glm: χ^2^ = 21.6, df = 1, *p* < .001), but the opposite was true for adulthood, where female mass loss was greater than male mass loss (glm: χ^2^ = 6.1, df = 1, *p* = .01; Tables S6, S7). Post hoc analyses examining pairwise comparisons between temperature treatment groups are given in Table S8.

### 3.4 High adult temperature and low body mass reduce starvation tolerance

**FIGURE 4:**
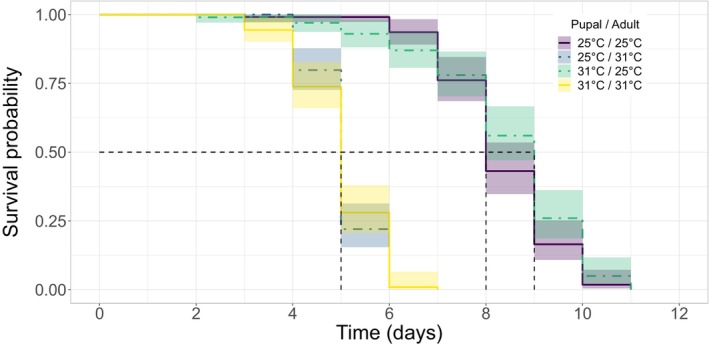
Probability of survival for unfed adult tsetse (*G. m. morsitans*) over time (days) under four temperature regimes throughout pupation and adulthood. Temperature was maintained at a constant 25°C or 31°C and is given as pupal/adult temperature. Matched pupal and adult temperature is depicted by solid lines and unmatched temperatures are depicted by dot‐dash lines. 95% confidence intervals are shaded and black dotted lines mark where 50% of the population has survived.

**FIGURE 5:**
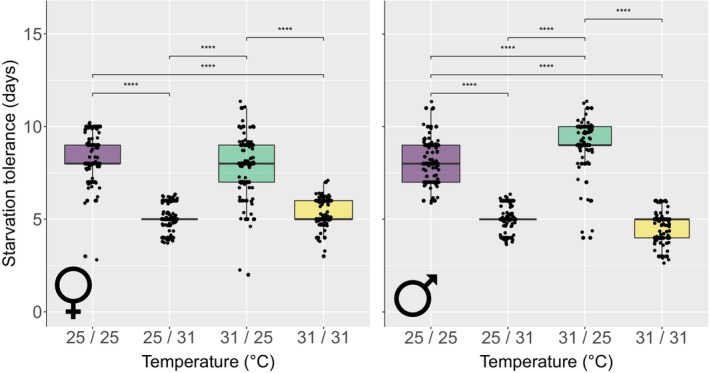
Female (♀) and male (♂) tsetse (*G. m. morsitans*) median starvation tolerance (days) over pupation and adulthood, under food deprivation, under four constant temperature regimes (pupal/adult) of 25°C or 31°C. Shown are the median, interquartile range and confidence interval (boxes and lines) as well as the individual data points. *p < .05; **p < .01; ***p < .001; ****p < .0001.

Starvation tolerance was primarily influenced by adult temperature, with 40% greater starvation risk in flies maintained at 31°C in comparison to those at 25°C, consequently reducing median survival time from 8 (IQR 7–9) to 5 (IQR 5–5.25) days (χ^2^ = 279, df = 3, *p* < .001; Table 1; Figure 4). There was no effect of experiencing matched pupal and adult temperature on tolerance, or evidence for negative carryover effects (Table 1). Pupae with initially greater mass had reduced starvation risk; for every additional 1 mg body weight, risk decreased by 16% (χ^2^ = 46.5, df = 1, *p* < .001; Table 1). We found a significant interaction between sex and temperature (χ^2^ = 31.7, df = 3, *p* < .001; Table 1; Figure 5; Figure S2a,b), whereby male tsetse took longer to starve than females when pupae were maintained at 31°C and adults at 25°C, yet starved more quickly than females when kept at 31°C for their entire lives. This is suggestive of a priming effect of being maintained at 31°C, for males only. Post hoc analysis examining the interaction between treatment temperature and sex is given in Table S9.

**TABLE 1:** Multivariate Cox proportional‐hazards models examining survival risk of unfed tsetse (*G. m. morsitans*) under four constant temperature regimes, given as pupal/adult temperature (°C; *N* = 428).

Variable	Treatment	Hazard ratio	Z‐score	*p*‐Value
Temperature	25/31	**45.97 (26.17–80.74)**	**13.32**	**<.001**
	31/25	1.30 (0.80–1.91)	1.36	.17
	31/31	**29.71 (17.39–50.75)**	**12.41**	**<.001**
Pupal mass		**0.85 (0.81–0.89)**	**−6.72**	**<.001**
Sex	Male	**1.23 (0.84–1.80)**	**1.09**	.28
Temperature × Sex	25/31 × Male	0.84 (0.50–1.44)	−0.62	.54
	31/25 × Male	**0.35 (0.20–**0.61**)**	**−3.70**	**<.001**
	31/31 × Male	**1.71 (0.99–2.94)**	**1.94**	**.05**

*Note*: Hazard ratios of greater than 1 suggest higher risk of death, and below 1 lower risk of death compared to the reference group. Significant results are in bold font.

**FIGURE 6:**
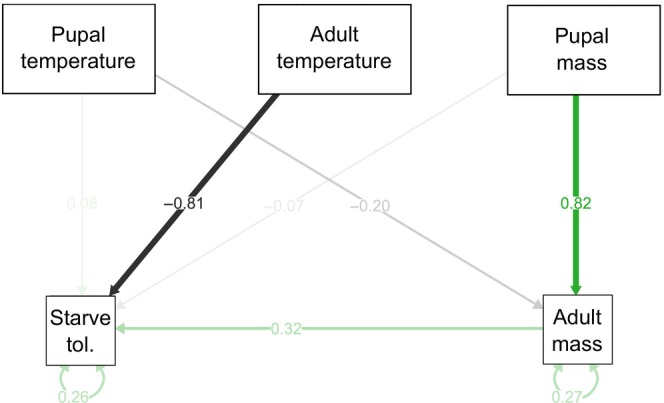
Path analysis showing the effect of mass and temperature on adult starvation tolerance (Starve tol.) in tsetse (*G. m. morsitans*). Straight arrows indicate a causal relationship. Variance is indicated by double‐ended curved arrows. The path coefficient is overlayed on each arrow and indicates the strength of each causal relationship. Higher colour intensity indicates stronger relationship weights, green for positive associations while black denotes negative associations.

Path analysis indicated that the largest effect on starvation tolerance was adult temperature, in accordance with the survival analysis (Table 2; Figure 6). Path analysis also illustrated an indirect effect of pupal temperature on starvation tolerance through its effect on adult mass. High pupal temperature produced flies with smaller adult mass which, in turn, show reduced starvation tolerance. Pupal mass was strongly related to adult mass illustrating the importance of maternal effects on starvation tolerance.

**TABLE 2:** Results from path analysis investigating the effect of pupal temperature and mass on adult mass, as well as the effect of pupal and adult temperature and mass on starvation tolerance (days) in tsetse (*G. m. morsitans*).

	Estimate	SE	Z‐value	*p*‐Value	Standardised latent coef.	Standardised coef.	*R* ^2^
Regressions							
Starvation tolerance ~							
Pupal temp	**0.05**	**0.02**	**2.9**	**.004**	**0.05**	**0.08**	.74
Adult temp	**−0.55**	**0.02**	**−32.5**	**<.001**	**−0.55**	**−0.81**	
Pupal mass	−0.07	0.04	−1.6	.11	−0.07	−0.08	
Adult mass	**0.27**	**0.04**	**6.7**	**<.001**	**0.27**	**0.32**	
Adult mass ~							
Pupal temp	**−0.16**	**0.02**	**−7.8**	**<.001**	**−0.16**	**−0.20**	.73
Pupal mass	**0.93**	**0.03**	**32.6**	**<.001**	**0.93**	**0.83**	
Variances							
Starvation tolerance	**1.09**	**0.08**	**14.6**	**<.001**	**1.09**	**0.26**	
Adult mass	**1.61**	**0.11**	**14.6**	**<.001**	**1.61**	**0.27**	

*Note*: Significant results are in bold font.

Abbreviation: SE, standard error.

## 4 DISCUSSION

Here, we found that starvation tolerance in tsetse was largely dependent on the temperature to which newly emerged (teneral) adults were exposed; teneral flies died 3 days earlier if kept at 31°C compared to 25°C. In contrast, pupal temperature played a minor or non‐existent role on starvation tolerance. Thus, in contrast to our predictions, we did not find strong evidence for adaptive developmental plasticity, as there was no effect of matching pupal and adult temperatures on starvation tolerance. Similarly, we did not find evidence of direct carryover effects—overall, a 6°C difference in pupal temperature did not affect adult starvation.

The largest effect was that of adult temperature on starvation tolerance, as found in other similar studies (Pijpe et al., 2007; Scharf et al., 2014). High temperatures elevate the metabolic rate of ectothermic species, thus energy reserves burn more rapidly. The pupal stage can mitigate some of the impacts of elevated temperatures due to its inactivity, as evidenced by a tenfold reduction in the rate of mass loss. A positive log‐linear relationship has been demonstrated in various tsetse species where adult metabolic rate increases between 20°C and 32°C (Terblanche et al., 2005). Correspondingly, fly feeding (i.e. bite rate) is predicted to double with a 5°C temperature increase (Terblanche & Chown, 2007). Therefore, as conditions become warmer, emerging tsetse will need to locate hosts rapidly or risk starvation. Additionally, feeding is a high‐risk activity, so a higher bite rate is likely to result in increased mortality of tsetse through predation as temperature increases (Hargrove & Williams, 1995; Randolf et al., 1992). Indeed, mark‐recapture studies conducted in Zimbabwe and Tanzania find that adult tsetse survival probability decreases with increasing temperature (Hargrove, 2001) and that mortalities in the field are particularly high in the hot season (see references within Hargrove, 2004). In Zimbabwe, temperature increases may already be causing declines in tsetse abundance. At Rekomitjie research station, tsetse numbers have been continually recorded since the 1960s and these data show a drastic decline in the numbers of adult flies collected (Mangwiro et al., 1999; Thomson, 1987; Torr et al., 1992). Mechanistic modelling suggests that the decline can be explained by a 0.9°C increase in temperature since 1975 (Lord et al., 2018).

We found some evidence of indirect carryover effects on starvation tolerance through the effect of development temperature on adult body mass. When pupae were maintained at 31°C, the size and mass of emerging adult flies was reduced. This result is found generally in insects (the temperature‐size rule, for example, Kingsolver & Huey, 2008) and in tsetse specifically (Bursell, 1960). Our path analysis also found that flies with lower mass had reduced starvation tolerance. Although not measured specifically in our experiment, this is likely due to reduced lipid and/or water reserves in smaller flies. The relationship between larger body size and improved starvation tolerance has been found in other insect species, such as aquatic bugs, beetles and mosquitoes (Gergs & Jager, 2014; Lehmann et al., 2006; Renault et al., 2003). Selective elimination of small tsetse has also been observed in field populations (Bursell & Glasgow, 1960; Jackson, 1948). A study by Phelps and Clarke (1974) found that field‐caught flies were significantly larger than adults from field‐collected pupae reared to adulthood in a laboratory setting. This discrepancy in size suggests there was selective elimination of small‐bodied flies in the hot season when the mortality of small flies was up to 76% (Phelps & Clarke, 1974). In this way, pupal temperature is indirectly related to starvation tolerance: high development temperatures produce small, light flies and flies of lower mass starve more quickly. However, it is important to note that, overall, this effect did not translate into greater starvation tolerance when pupae were developed at 31°C.

In contrast, there was some evidence of adaptive priming causing greater starvation tolerance when males were maintained at 31°C for development, but this was only apparent when these males were transferred to 25°C for adulthood. This improved ability to survive longer without a blood meal when exposed to high developmental temperature could be due to plasticity, or acclimation, causing changes to cell stress responses and repair processes (Sørensen et al., 2003). A similar effect was found in a study on red flour beetles where a high development temperature allowed adults to survive for longer when starved (Scharf et al., 2014). For the beetles, this effect was present at both high and low adult temperatures, and in both sexes, but was more pronounced when adult starvation was tolerated at low temperature. Another study on *B. anynana* also found that butterflies reared at high temperatures were more starvation resistant, particularly when adults were starved at low temperature (Pijpe et al., 2007). In contrast to our study, the authors found starvation tolerance was higher in female butterflies. These findings could indicate an adaptive response of developmental stages to high temperature, where effects are only apparent when adults are kept at a benign temperature. Alternatively, in the present study, there may have been selective disappearance of less tolerant individuals in the 31°C treatment. Of the 3% of flies which failed to emerge successfully overall, more belonged to the 31°C treatment (*N* = 10/220: 4.5%) compared to 25°C (*N* = 2/220: 0.9%), so the removal of these less tolerant individuals could have caused a higher average starvation tolerance in the remainder. Assuming a 1:1 sex ratio, it is likely that these individuals were largely male, as of the emerged adult flies, *N* = 222 were female and *N* = 206 were male – explaining why this effect may have only been apparent in males.

Adaptive developmental plasticity can evolve where there is environmental variability, high environmental predictability and low cost of plastic response (Monaghan, 2008). As pupal and adult tsetse ecology are so different – highly mobile, flying adults versus immobile pupae, deposited in sheltered microclimates (Buxton, 1955) – pupal temperature may not adequately predict adult conditions. These distinctive ecologies could be why we found no effect of matching temperature conditions in this experiment. Future work could consider the effect of in‐utero exposure to higher temperatures instead (during the 9 days when larvae are developing within their mother), which may be more predictive of adult temperature conditions. Indeed, higher pupal mass improved starvation tolerance indicating the importance of maternal provisioning given that the only nutrients provided to the larva in tsetse come from the mother in utero. Additionally, the high‐temperature treatment in this experiment may have killed symbionts of tsetse, which are important for the maintenance of homeostasis (Michalkova et al., 2014). *Wigglesworthia glossinidia* (present in all flies) and *Sodalis glossinidius* (not obligate) are maternally transferred endosymbionts that colonise tsetse tissues (Wang et al., 2013). Studies have shown that heat tolerance, longevity and/or fecundity are reduced when these bacterial symbionts are pharmaceutically eliminated (Dale & Welburn, 2001; Pais et al., 2008). Moreover, *Sodalis glossinidius* does not grow at temperatures above 31°C and is unable to survive for more than 48 h at 30°C (Roma et al., 2019). These bacteria were likely killed in the 31°C treatments and may have influenced the ability of tsetse to respond adaptively. Future investigations into how symbiont populations are affected by temperature in mother and offspring flies would give insight into the role of these symbionts in mediating survival responses to high temperatures.

Overall, we find that adult temperature had the most pronounced effect on starvation tolerance in tsetse. Pupal temperature had non‐existent or opposing effects, showing that starvation responses to temperature are life stage specific. We found no convincing evidence of adaptive developmental plasticity or negative carryover effects in tsetse. Therefore, rising temperatures due to climate change are likely to exert the greatest effect on emerging adult tsetse, causing metabolic rate to increase and forcing flies to locate their first blood meal more rapidly.

## AUTHOR CONTRIBUTIONS

**Hester Weaving:** Conceptualization (supporting); data curation (lead); formal analysis (lead); funding acquisition (supporting); methodology (supporting); writing – original draft (lead); writing – review and editing (lead). **Jennifer S. Lord:** Conceptualization (equal); methodology (equal); writing – original draft (supporting); writing – review and editing (supporting). **Lee Haines:** Conceptualization (equal); methodology (equal); writing – original draft (supporting); writing – review and editing (supporting). **Sinead English:** Conceptualization (equal); funding acquisition (lead); methodology (equal); supervision (lead); writing – original draft (supporting); writing – review and editing (supporting).

## CONFLICT OF INTEREST STATEMENT

The authors certify that they have no conflicts of interest.

## Supporting information


**Table S1.** **–S9.**
Figure S1.–S2.

## Data Availability

Data and code are published on the OSF database: https://osf.io/t4r5s/.
