# Supplementary material for: No evidence for direct thermal carryover effects on starvation tolerance in the obligate blood‐feeder, Glossina morsitans morsitans
Source: Ecol Evol. 2023 Oct 18;13(10):e10652. doi: 10.1002/ece3.10652 (PMC10585125; doi:10.1002/ece3.10652)
Supplement: Supplementary file 1 — Table S1. –S9. Figure S1.–S2. [file ECE3-13-e10652-s001.docx]

**Supplementary Information**

**Supplementary Table 1** Mean mass (mg) of tsetse (*G. m. morsitans*) pupae from different maternal age groups (weeks). Mean differences between groups are given ± standard deviation (SD). ANOVA was used to compare differences between groups with full statistics (adjusted R^2^; F statistic; degrees of freedom) displayed below the comparison statistics.

| Mother age (weeks) | Mass (mg) ± SD | Mean difference ± SD | T-value | P-value |
| --- | --- | --- | --- | --- |
| *Reference* | 25.35 ± 0.21 |  |  |  |
| 4 - 5 | **26.80 ± 0.19** | **1.45 ± 0.28** | **5.19** | **<0.001** |
| 6 - 7 | **27.07 ± 0.18** | **1.72 ± 0.27** | **6.27** | **<0.001** |
| 8 - 9 | **26.34 ± 0.22** | **0.99 ± 0.30** | **3.30** | **0.001** |
| Adj. R^2^ = 0.085; F = 14.6; df = 3, 436 | | | | |

**Supplementary Table 2** Pupal development time (mean ± standard deviation (SD)) of female and male tsetse (*G. m. morsitans*) under two constant temperature regimes: 25°C and 31°C.

| Temperature (°C) | Sex | N | Development time ± SD (days) |
| --- | --- | --- | --- |
| 25 | Female | 109 | 30.61 ± 2.78 |
|  | Male | 109 | 32.12 ± 2.81 |
| 31 | Female | 113 | 19.64 ± 0.55 |
|  | Male | 97 | 21.40 ± 0.59 |

**Supplementary Table 3** Generalised Least Squares model examining the effect of constant 25°C or 31°C on pupal development time (days) in tsetse (*G. m. morsitans*). Variances were assumed to be unequal across temperature treatments. All pupae that emerged were considered in the analysis. Significant results are in bold font. Comparisons are made with the reference group and results are back-transformed from the log scale.

| Variable (reference group) | Estimate (95% CIs) | T-value | P -value |
| --- | --- | --- | --- |
| Intercept | **31.66 (30.20 - 33.19)** | **144.0** | **<0.0001** |
| Pupal temperature (25) | **0.64 (0.63 - 0.66)** | **-47.4** | **<0.0001** |
| Sex (female) | **1.05 (1.02 - 1.08)** | **3.9** | **0.0001** |
| Pupal mass | 1.00 (1.00 - 1.00) | -1.7 | 0.09 |
| Temp x sex | **1.04 (1.01 - 1.06)** | **2.7** | **0.006** |

| Comparison | Estimate | SE | df | T-value | P -value |
| --- | --- | --- | --- | --- | --- |
| 25/female - 31/female | **0.440** | **0.009** | **252** | **47.4** | **<0.0001** |
| 25/female - 25/male | **-0.049** | **0.013** | **216** | **-3.9** | **0.0008** |
| 25/female - 31/male | **0.354** | **0.009** | **259** | **37.9** | **<0.0001** |
| 31/female - 25/male | **-0.488** | **0.009** | **252** | **-52.6** | **<0.0001** |
| 31/female - 31/male | **-0.085** | **0.004** | **208** | **-22.2** | **<0.0001** |
| 25/male - 31/male | **0.403** | **0.009** | **259** | **43.1** | **<0.0001** |

**Supplementary Table 4** Post-hoc analysis examining the interaction between sex and pupal temperature on development time. The Tukey method was used for P value adjustment. Results have not been back transformed from the log scale.

**Supplementary Table 5** Linear models examining the effect of pupal temperature (constant 25°C or 31°C), sex and maternal age on adult mass (mg) and wing length (mm) in tsetse (*G. m. morsitans*). Mean differences between groups are given ± standard deviation (SD). Full statistics (adjusted R^2^; F statistic; degrees of freedom) are displayed for each model below the comparison statistics.

|  |  | Wing vein (mm) | | | |  | Adult mass (mg) | | | |
| --- | --- | --- | --- | --- | --- | --- | --- | --- | --- | --- |
| **Variable** | **Group** |  | **Estimate ± SD** | **T-value** | **P-value** |  |  | **Estimate ± SD** | **T-value** | **P-value** |
| Intercept |  |  | **1.61 ± 0.007** | **224.0** | **<0.0001** |  |  | **22.09 ± 0.27** | **81.4** | **<0.0001** |
| Pupal temp. (25) | 31 |  | **-0.021 ± 0.006** | **-3.53** | **0.0004** |  |  | **-1.19 ± 0.22** | **-5.39** | **<0.0001** |
| Sex (female) | Male |  | **-0.16 ± 0.006** | **-27.3** | **<0.0001** |  |  | **-0.79 ± 0.22** | **-3.59** | **0.0003** |
| Maternal age (weeks) | 4 - 5 |  | 0.013 ± 0.008 | 1.54 | 0.12 |  |  | **1.43 ± 0.31** | **4.57** | **<0.0001** |
|  | 6 - 7 |  | 0.015 ± 0.008 | 1.80 | 0.07 |  |  | **1.71 ± 0.31** | **5.57** | **<0.0001** |
|  | 8 - 9 |  | -0.0019 ± 0.009 | -0.21 | 0.83 |  |  | **0.81 ± 0.33** | **2.43** | **0.015** |
|  |  | Adj. R^2^ = 0.66; F = 151.8; df = 379, 5 | | | |  | Adj. R^2^ = 0.14; F = 14.87; df = 422, 5 | | | |

**Supplementary Table 6** Mean mass ± standard deviation (SD) (mg) loss of tsetse (*G. m. morsitans*) during pupation and adulthood. Pupae were kept at either constant 25°C or 31°C for development and transferred to either a matched or unmatched temperature upon emergence for adulthood, given as pupal / adult temperature.

| **Variable** | **Group** | **N** | **Pupal mass loss ± SD (mg)** | **Pupal mass loss rate (mg/day)** | **Adult mass loss ± SD (mg)** | **Adult mass loss rate (mg/day)** |
| --- | --- | --- | --- | --- | --- | --- |
| Temperature | 25 / 25 | 109 | 3.78 ± 4.72 | 0.12 | 8.8 ± 1.92 | 1.065 |
|  | 25 / 31 | 109 |  |  | 11.0 ± 2.76 | 2.187 |
|  | 31 / 25 | 103 | 4.75 ± 1.36 | 0.23 | 8.9 ± 2.63 | 1.059 |
|  | 31 / 31 | 107 |  |  | 10.4 ± 2.93 | 2.087 |
| Sex | Female | 222 | 4.00 ± 1.35 | 0.16 | 10.1 ± 2.79 | 1.519 |
|  | Male | 206 | 4.53 ± 1.36 | 0.17 | 9.5 ± 2.66 | 1.423 |

|  |  |  |  |  |  |
| --- | --- | --- | --- | --- | --- |


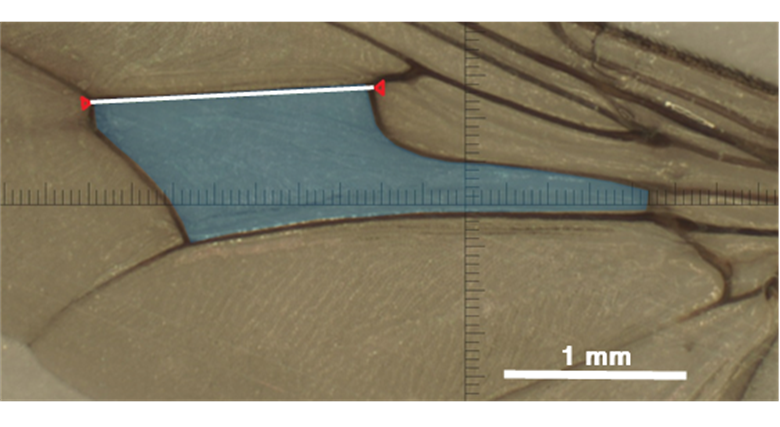


**Supplementary Figure 1** Ventral view of tsetse (*G. m. morsitans*) wing with hatchet cell highlighted in blue. The length of the upper vein of the hatchet cell, marked between two red arrows, was taken as a proxy for size.

**Supplementary Table 7** Multivariate General Linear Models examining the effect of four temperature regimes and sex on pupal and adult mass loss in unfed tsetse (*G. m. morsitans*). Pupae were kept at either constant 25°C or 31°C for development and transferred to either a matched or unmatched temperature upon emergence for adulthood, given as pupal / adult temperature. Mean differences between groups are given ± standard deviation (SD). Full statistics (adjusted R^2^; F statistic; degrees of freedom) are displayed for each model below the comparison statistics.

|  |  |  | Pupal | | |  | Adult | | |
| --- | --- | --- | --- | --- | --- | --- | --- | --- | --- |
| **Variable** | **Group** | **N** | **Mean difference ± SD** | **T-value** | **P-value** |  | **Mean difference ± SD** | **T-value** | **P-value** |
| Temperature | 25 / 25 | 109 | *Reference* |  |  |  | *Reference* |  |  |
|  | 25 / 31 | 109 |  |  |  |  | **2.15 ± 0.35** | **6.17** | **<0.001** |
|  | 31 / 25 | 103 |  |  |  |  | 0.04 ± 0.35 | 0.12 | 0.91 |
|  | 31 / 31 | 107 | **0.25 ± 0.029** | **8.56** | **<0.001** |  | **1.52 ± 0.35** | **4.35** | **<0.001** |
| Sex | Female | 222 | *Reference* |  |  |  | *Reference* |  |  |
|  | Male | 206 | **0.15 ± 0.029** | **5.01** | **<0.001** |  | **-0.61 ± 0.25** | **-2.47** | **0.014** |
|  |  |  | Adj. R^2^ = 0.18; F = 47.6; df = 425, 2 | | |  | Adj. R^2^ = 0.12; F = 15.7; df = 423, 4 | | |

**Supplementary Table 8** Post-hoc analysis examining the effect of four temperature regimes on total mass loss over pupation and adulthood in unfed tsetse (*G. m. morsitans*). Temperature is given as pupal / adult. Pairwise comparison mean differences between groups are given with standard error (SE). The Tukey method was used for P value adjustment. Significant differences are in bold font.

| Comparison | Estimate | SE | df | T-value | P-value |
| --- | --- | --- | --- | --- | --- |
| 25 / 25 - 25 / 31 | **-2.01** | **0.35** | **424** | **-5.72** | **<0.0001** |
| 25 / 25 - 31 / 25 | **-0.94** | **0.36** | **424** | **-2.65** | **0.04** |
| 25 / 25 - 31 / 31 | **-2.46** | **0.35** | **424** | **-6.99** | **<0.0001** |
| 25 / 31 - 31 / 25 | **1.06** | **0.36** | **424** | **2.99** | **0.02** |
| 25 / 31 - 31 / 31 | -0.46 | 0.35 | 424 | -1.30 | 0.57 |
| 31 / 25 - 31 / 31 | **-1.52** | **0.36** | **424** | **-4.26** | **0.0002** |


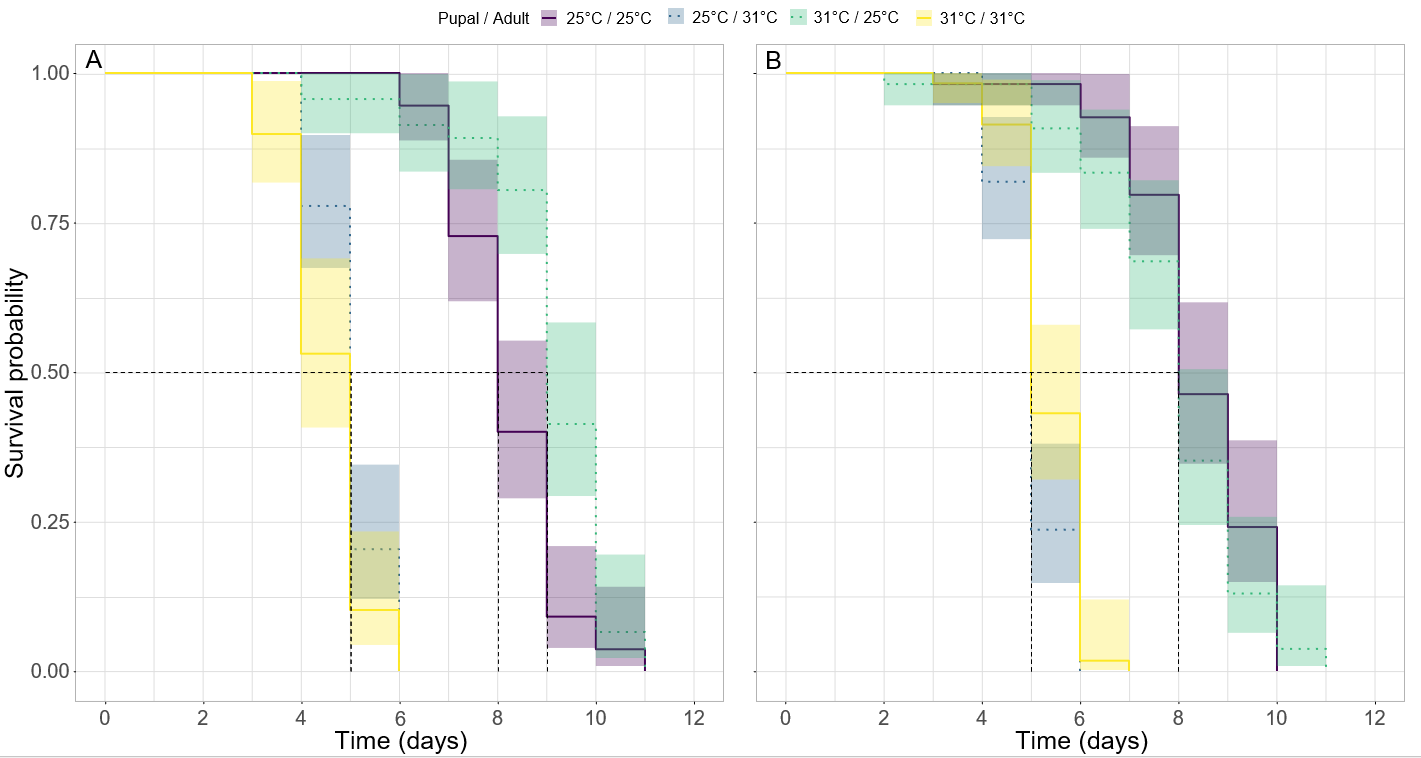


**Supplementary Figure 2** Probability of survival of A.) male and B.) female adult tsetse (*G. m. morsitans*) over time (days) in absence of blood meals under four temperature regimes throughout pupation and adulthood. Temperature was maintained at constant 25°C or 31°C and are given as pupal / adult temperature. Matched pupal and adult temperature is depicted by solid lines and unmatched temperatures are depicted by dot-dash lines. 95% confidence intervals are shaded and black dotted lines mark where 50% of the population have survived.

**Supplementary Table 9** Post-hoc comparison for interaction between treatment temperature and sex for Multivariate Cox Proportional-Hazards Model examining survival risk of tsetse (G. m. morsitans) when deprived of blood meals under four constant temperature regimes. Temperatures are given as pupal / adult. Pairwise comparison mean differences between groups are given with standard error (SE). The Tukey method was used for P value adjustment. Significant differences are in bold font.

| Comparison | Estimate | SE | Z-ratio | P-value |
| --- | --- | --- | --- | --- |
| 25 / 25 female - 25 / 31 female | **-3.83** | **0.29** | **-13.3** | **<0.0001** |
| 25 / 25 female - 31 / 25 female | -0.26 | 0.20 | -1.4 | 0.88 |
| 25 / 25 female - 31 / 31 female | **-3.39** | **0.27** | **-12.4** | **<0.0001** |
| 25 / 25 female - 25 / 25 male | -0.21 | 0.19 | -1.1 | 0.96 |
| 25 / 25 female - 25 / 31 male | **-3.87** | **0.29** | **-13.5** | **<0.0001** |
| 25 / 25 female - 31 / 25 male | 0.58 | 0.20 | 2.8 | 0.08 |
| 25 / 25 female - 31 / 31 male | **-4.14** | **0.29** | **-14.2** | **<0.0001** |
| 25 / 31 female - 31 / 25 female | **3.56** | **0.28** | **12.6** | **<0.0001** |
| 25 / 31 female - 31 / 31 female | 0.44 | 0.19 | 2.3 | 0.30 |
| 25 / 31 female - 25 / 25 male | **3.62** | **0.28** | **12.8** | **<0.0001** |
| 25 / 31 female - 25 / 31 male | -0.04 | 0.19 | -0.2 | 1 |
| 25 / 31 female - 31 / 25 male | **4.40** | **0.30** | **14.6** | **<0.0001** |
| 25 / 31 female - 31 / 31 male | -0.31 | 0.20 | -1.6 | 0.77 |
| 31 / 25 female - 31 / 31 female | **-3.13** | **0.27** | **-11.6** | **<0.0001** |
| 31 / 25 female - 25 / 25 male | 0.05 | 0.19 | 0.3 | 1 |
| 31 / 25 female - 25 / 31 male | **-3.60** | **0.28** | **-12.7** | **<0.0001** |
| 31 / 25 female - 31 / 25 male | **0.84** | **0.20** | **4.1** | **0.001** |
| 31 / 25 female - 31 / 31 male | **-3.87** | **0.29** | **-13.4** | **<0.0001** |
| 31 / 31 female - 25 / 25 male | **3.18** | **0.27** | **11.9** | **<0.0001** |
| 31 / 31 female - 25 / 31 male | -0.48 | 0.19 | -2.5 | 0.20 |
| 31 / 31 female - 31 / 25 male | **3.97** | **0.29** | **13.8** | **<0.0001** |
| 31 / 31 female - 31 / 31 male | **-0.75** | **0.20** | **-3.8** | **0.004** |
| 25 / 25 male - 25 / 31 male | **-3.66** | **0.28** | **-12.9** | **<0.0001** |
| 25 / 25 male - 31 / 25 male | **0.79** | **0.20** | **3.9** | **0.003** |
| 25 / 25 male - 31 / 31 male | **-3.93** | **0.29** | **-13.7** | **<0.0001** |
| 25 / 31 male - 31 / 25 male | **4.45** | **0.30** | **14.7** | **<0.0001** |
| 25 / 31 male - 31 / 31 male | -0.27 | 0.20 | -1.4 | 0.88 |
| 31 / 25 male - 31 / 31 male | **-4.71** | **0.31** | **-15.5** | **<0.0001** |
